# “And just like that, quiet”: a content analysis of TikTok videos on food noise

**DOI:** 10.1038/s41387-026-00423-z

**Published:** 2026-04-29

**Authors:** Daisuke Hayashi, Janelle Kort, Isabel M. Robles-Martinez, Diana Orabueze, Katrina Bakhl, Travis D. Masterson

**Affiliations:** 1 Department of Nutritional Sciences, College of Health and Human Development, The Pennsylvania State University, University Park, PA USA; 2 Department of Psychological Sciences: Health Psychology, University of California, Merced, CA USA; 3 Penn State College of Medicine, Hershey, PA USA

**Keywords:** Nutrition, Patient education

## Abstract

**Background:**

Food Noise is a topic of growing interest in media, social media, and reports from patients and clinicians. A theoretical definition of “Food Noise” has recently been established as “heightened and/or persistent manifestations of food cue reactivity, often leading to food-related intrusive thoughts and maladaptive eating behaviors,” but research focusing on the lived experiences of people who report experiencing it is limited. TikTok has become a major outlet for content creators to disseminate information on Food Noise, with over 3600 videos under the hashtag #FoodNoise as of June 2024.

**Objectives:**

This study aimed to examine the top videos on TikTok under the hashtag “FoodNoise” and explore what content creators discuss around food noise.

**Methods:**

We analyzed 100 videos on TikTok under the hashtag #FoodNoise. Video links and metadata (such as engagement metrics) were retrieved on June 24^th^, 2024. After one duplicated video was excluded, the final analysis included 99 videos. Following pilot testing of the codebook, we conducted a quantitative content analysis of the videos. This study required no ethical approval.

**Results:**

The sampled videos had a mean of 1,173,323.63 views, 8,154.57 likes, 246.99 comments, and 582.65 shares. Content creators were primarily female (91.92%), aged 30 or older (82.83%), and White (85.86%). 22.22% of content creators were healthcare professionals, and 70.71% of videos were patient testimonies. 49.49% of videos mentioned medications, mainly GLP-1 receptor agonists. 42.42% mentioned food, mostly candies, desserts, and fast foods. Of the videos that defined Food Noise (82.82%), 93.9% defined it consistently with the current theoretical definition. Most videos (85.86%) depicted food noise negatively.

**Conclusion:**

The top content available on TikTok on food noise is mainly comprised of patient testimonies that describe food noise as negative and distressful, and depict the use of medications, mostly GLP-1RAs, as a positive strategy to help manage food noise.

## Introduction

The term food noise has recently emerged in anecdotal reports from patients and clinicians and has been widely discussed on major media outlets and social media to describe the experiences of people who report constantly thinking about food and eating, to the point that they might feel like their lives “revolve around food” [1]. These obsessive thought patterns and ruminations about food have been theorized to be intense manifestations of food cue reactivity, leading to food-related intrusive thoughts and maladaptive eating behaviors [1]. The rapid increase in online searches for the term food noise can be observed in Google Trends data, which shows initial interest in the topic early in 2023 and an all-time high interest score during August 2025 in the US, and April 2025 worldwide [2], suggesting a continuous rise in online searches for the term until the moment of writing this paper. This trend is accompanied by the emergence in the use of the term food noise to market obesity medications and behavioral treatments, which promise to help patients “silence” or “quiet” their food noise through the use of medications [3, 4]. This includes medications such as GLP-1RAs, including semaglutide, which has been approved by the FDA for use in weight management in adults since June 2021 and pediatric patients aged 12 or older since December 2022 [5]. Despite the rapid spread in the use of the term food noise, which could prove useful to help patients describe their experiences and identify the need for interventions targeting maladaptive cognitions around food and eating, systematic research exploring food noise as a separate construct is in its early stages.

Recently, different authors have proposed theoretical definitions for food noise that could help explain food noise and guide future research beyond anecdotal reports. The distinction between such definitions has sparked scholarly discussion [6]. Hayashi and colleagues provided the earliest definition of food noise as “heightened and/or persistent manifestations of food cue reactivity, often leading to food-related intrusive thoughts and maladaptive eating behaviors,” a definition that theorizes that experiences of food noise are dynamic, and result from intense and persistent responses to internal and external food cues, likely due to a combination of constant and transient influencers ranging from genetic makeup and appetitive traits to changes in levels of appetite-regulating hormones, which are detailed in the Cue-Influencer-Reactivity-Outcome (CIRO) model of food cue reactivity [1]. Later, Diktas and colleagues [7], followed by Dhurandhar and colleagues [8], presented alternative definitions, both arguing against Hayashi’s definition’s mention of the role of reactivity to internal and external cues as precedents of experiences of food noise. For instance, Diktas and colleagues define food noise as “persistent, intrusive thoughts about food that are disruptive to daily life and make healthy behaviors difficult” [7], while Dhurandhar and colleagues described it as “Persistent thoughts about food that are perceived by the individual as being unwanted and/or dysphoric and may cause harm to the individual, including social, mental, or physical problems” [8]. All three definitions and how they are further detailed in the respective publications share fundamental aspects, such as the intrusive nature of food noise and its potential negative impact on health and well-being, and offer a helpful starting point to explain food noise from a theoretical perspective based on pre-existing research. However, it is essential to acknowledge that all three are arguably working definitions, coming from researchers, clinicians, and specialists in human eating behavior, to explain a phenomenon of which little is known beyond anecdotal reports, and which demands future research with diverse methodological approaches [6] to refine its understanding.

Given the rise in popularity of the term in digital spaces, social media offers an ideal platform for investigating what kind of content is being produced on the topic of food noise, both to further understand what individuals who claim to experience it may mean by food noise and to examine the nature of the information being offered to social media users on the topic. The widespread use and understanding of terms such as food noise could potentially lead to changes in consumer attitudes and behaviors around food, eating, and medical treatments, likely resulting in significant public health impacts. TikTok is a major social media platform where internet users share information, personal experiences, and (often undisclosed) sponsored content marketing products and services [9]. Boasting a viewership estimated to be 1.7 billion worldwide, 55% of whom are aged between 18 and 24, TikTok is currently one of the most successful and popular social media platforms, especially among adolescents and emerging adults [10, 11]. Initially created as a music-based entertainment app, it has now become a major outlet for topics such as news, politics, health, and social issues, as well as a means to quickly disseminate information, ideas, and beliefs [10, 12]. It allows users to create and consume short videos, which are presented based on an algorithm that tailors content to users’ indicated preferences and the content with which they engage within the platform [13]. Among the niches of “tailored” content within the platform, “MedTok” is a sub-section of the application specific to medicine-related content [14]. While medical content was present on TikTok prior to 2020, the COVID-19 pandemic led to an increase in users turning to the platform to obtain public health information regarding the virus. Since then, medical information has increased, covering a broad scope of illness and disease, with an increasing number of users turning to it, too [15]. As of 2020, the hashtags “medicine” and “doctor” had 1.4 billion and 6.7 billion views on TikTok, respectively, and do not account for the numerous other hashtags that have been used for other medically related videos [16]. A survey of 2000 people done by CharityRx, a prescription savings company, found that 1 in 5 people go to TikTok for medical advice before going to their physician. Additionally, of those who participated and were part of Gen Z, 1 in 3 mentioned TikTok as a major source of health information [15]. Given TikTok’s prominence as a platform where users seek and share health-related information and personal experiences, it offers a valuable opportunity to study the characteristics of the content being shared within the platform on food noise. Therefore, this study aimed to characterize a sample of the content available on TikTok on food noise, exploring what content creators define as food noise, the topics associated with it, and the characteristics of content creators.

## Methods

### Video sampling

An initial sample of 100 videos was obtained by entering the hashtag “#foodnoise” as a search term on TikTok’s search bar on the 24^th^ of June 2024. The top 100 videos retrieved by the search were included. One duplicated video was identified and excluded, resulting in 99 videos being included in the final analysis. This number of videos is reasonable considering previous data suggests most Internet users limit their content consumption to that within the first three pages of search results, which, in similar platforms, like YouTube, translates to approximately 60 videos [17]. We used the #foodnoise hashtag without additional search terms to avoid limiting our search to particular associated topics. This allowed us to identify what topics content creators associated with food noise in the top videos on the platform without defining such themes beforehand or limiting them to what has been described in anecdotal reports (e.g., GLP-1RA medications, dieting, weight loss, etc.) [1]. This study required no ethical approval since no human participants were involved, no identifiable private data was collected, and all videos evaluated were publicly available. All methods were performed in accordance with the relevant guidelines and regulations.

### Codebook development and data collection

The research team developed a codebook for this analysis over several meetings. In these meetings, team members viewed and discussed ten videos, focusing on specific factors that could be explored through content analysis. After familiarization with the content of the videos, the team agreed upon the following overarching questions, which guided the development of the codebook: How do content creators describe the concept of food noise? What other topics are discussed in videos about food noise? What are the demographic characteristics of the content creators? What is the apparent goal of the video? And which communication strategies are employed by the content creator? The development of the codebook was an iterative process, with multiple versions being tested and modified by having two coders provide ratings for the first 10 videos and then meeting with the lead author of this paper until the two coders fully agreed on the variables of interest and an adequate inter-rater reliability was reached. This iterative team-based process for codebook development was consistent with previous research on social media platforms [18] and combined the use of inductive and deductive reasoning.

The resulting codebook contained mostly multiple-choice questions that coded the variables of interest into discrete categories. For example, the professions declared by content creators were dichotomized into medical or healthcare professionals vs. non-medical or healthcare professionals. Similarly, the definitions and examples provided in the videos around the construct of food noise were coded as either consistent with the theoretical definition proposed by Hayashi et al. (i.e., “heightened and/or persistent manifestations of food cue reactivity, often leading to food-related intrusive thoughts and maladaptive eating behaviors”) [1] or inconsistent with such a definition. Definitions and examples were coded as “consistent with Hayashi et al.’s definition” (which was the only one available at the time when analyses were conducted) if they described the food-related cognitions and cravings that occur in food noise as having any combination of the following characteristics: heightened (e.g., describing them as too intense, overwhelming, or salient), persistent (e.g., describing thinking about food all the time or for long periods of time), intrusive (e.g., describing thinking of food when trying to focus on something else or being distressed by such thoughts), and leading to maladaptive eating behaviors (e.g., reporting that such thoughts result in overeating, loss of control over eating, or feeling guilty about one’s eating). Definitions or examples that contradicted any of these aspects were coded as “inconsistent with Hayashi et al.’s definition”.

Text-input fields were added so that coders could transcribe quotes that primarily served to exemplify certain variables of interest (e.g., definitions or experiences related to food noise reported by the content creators). Additional open-text fields were used to identify foods and medications mentioned in the videos. The final codebook (fully detailed in Appendix A) comprised 17 multiple-choice questions and 10 text-input fields capturing the following variables of interest: perceived (i.e., observed by the coder) content creator demographics (age group, sex, race, profession stated in the video or content creator’s profile), presence of a sponsorship statement, mentions of scientific literature as a source of information, communication strategies employed by content creator, perceived purpose of the video, the content creator’s definition of food noise, and whether other topics of interest to our research were mentioned in the video. If a topic of interest was mentioned in the video, the coders also documented if such mentions included testimonies, explicit advice, and a visual depiction. Due to the frequent mention to medications and commercial products to manage food noise in the ten videos included in the pilot analysis, the codebook also included codes for the perceived attitude depicted by the content creators toward food noise and the use of medications, compound medications, or herbal and nutritional supplements to manage it as positive (univalent positive attitude, such as exclusively mentioning good things about the topic), negative (univalent negative attitude, such as exclusively mentioning bad things about the topic), ambivalent (torn or uncertain attitude, such as listing pros and cons or internal conflict towards the topic), or neutral (attitudes that were neither positive or negative, or that were indifferent towards the topic). Coders were instructed to examine the tone, language, and visual cues expressing the creator’s attitude toward a topic of interest. Coders also collected video reach and engagement data (i.e., views, likes, comments, shares, and bookmarks), video duration (in seconds), the number of followers of content creators, and the hashtags used in the videos other than #FoodNoise. View count was not available for 31 videos, and thus was computed as missing data and removed from the calculation of the mean number of views. At the time of data collection, TikTok displayed engagement metrics with values equal to or greater than ten thousand, rounded to the nearest thousand, and equal to or greater than one million, rounded to the nearest million. Coders entered data on the Research Electronic Data Capture (REDCap) program hosted at Penn State Health Milton S. Hershey Medical Center and Penn State College of Medicine. REDCap is a secure, web-based application that supports data capture for research studies [19].

The primary researcher (DH), who established the initial research question, provided oversight during the development of the codebook and criteria for this quantitative content analysis, and had a primary role in resolving inter-coder disagreements and interpreting the data, identifies as a mixed-race male of Latin American origin, a behavioral researcher, and a registered dietitian with previous experience in providing nutrition counseling to patients with obesity and chronic diseases.

### Coding reliability

To achieve intercoder reliability, we adopted a standard of at least 80% agreement across at least 95% of the code categories [20]. Initially, coders worked by coding the first ten videos from the list until these criteria were met. After multiple iterations of the codebook and meetings between the two coders and the first author, an overall agreement rate between coders of 95.3% was reached. 96.36% of codes achieved an inter-rater agreement above 80%. The coding of the remaining 89 videos was then split between the two coders.

### Descriptive statistics

Quantitative data were reported as frequencies, and each video was treated as a unit of analysis. Summary statistics for each coded variable and engagement metrics were generated on IBM SPSS Statistics for Windows, Version 29.0 (IBM Corp., Armonk, NY, USA). Frequency of specific stated professions and the mentions of different categories of foods and medications were tallied, and frequencies of hashtags were visualized as a word cloud using NVivo 14 (Lumivero, Denver, CO, USA).

## Results

Of the content creators who appeared in the videos, 91 were coded as female, 82 as adults aged 30 or older, and 85 as White. A total of 77 were not healthcare professionals (Table 1). 17 out of 99 content creators were emerging adults, and none were adolescents. The most frequent healthcare professions declared by content creators were medical doctors across numerous specialties (*n* = 8), registered dietitians (*n* = 7), and personal trainers/fitness coaches (*n* = 3). One content creator stated being a nurse practitioner, and another declared being a nutritional counselor. Engagement and reach metrics are presented in Table 2. Mean video duration was 97.31 s (SD = 90.183), ranging from 6 to 578 s.

**Table 1:** Perceived demographics and stated profession of TikTok content creators posting about “Food Noise” (*n* = 99).

Variable	Category	n	%
Perceived age group	Adolescent (13–17)	0	0.0
	Emerging adult (18–29)	17	17.17
	Adult (30 + )	82	82.83
Perceived sex	Female	91	91.92
	Male	8	8.08
Perceived race	White	85	85.86
	Non-White	14	14.14
Stated profession	Healthcare professional	22	22.22
	Other	77	77.78

All demographics are based on perceived characteristics unless explicitly stated in the video. Content creator profiles were consulted for stated profession if not present in the video. Percentages are rounded and may not sum to exactly 100%.

**Table 2:** Engagement metrics of TikTok videos about “Food Noise” (*n* = 99).

Metric	Minimum	Maximum	Mean	SD
Followers	99	2,400,000	135,216.23	268,641.28
Views	68	15,000,000	1,173,323.63	3,152,316.56
Likes	57	193,600	8,154.57	26,701.84
Comments	1	2,806	246.99	530.17
Bookmarks	2	16,900	660.78	2,198.73
Shares	0	21,600	582.65	2,532.48

Metrics reflect publicly available reach and engagement indicators per video. “SD” = standard deviation. All variables are based on the 99 valid videos analyzed. The number of “followers” is related to the content creators’ profile pages.

Of the 99 videos analyzed, 70 included patient testimonies, 54 aimed to inform the viewer, and 85 depicted food noise negatively (Table 3). A total of 94 videos provided some form of definition of food noise. Of these, 77 were coded as consistent with the definition proposed by Hayashi et al., which describes food noise as “heightened and/or persistent manifestations of food cue reactivity, often leading to food-related intrusive thoughts and maladaptive eating behaviors.” Five videos (5.05%) provided definitions inconsistent with this framework, four of which characterized food noise as normal physiological hunger cues. We have included below illustrative quotes extracted from videos, one consistent with Hayashi et al.’s definition and one inconsistent.

*“Food noise is these all-consuming, relentless, intrusive thoughts about food. It’s this overwhelming preoccupation about what to eat, when to eat, and how to satisfy cravings. This is different from physical hunger. This happens regardless of physical hunger. People who have food noise feel like their lives revolve around food.” – Illustrative quote consistent with Hayashi and colleagues’ definition of food noise*.

*“I think food noise is just hunger, appetite, and craving. It’s not a bug, it’s a feature of human life and human bodies.”* - Illustrative quote inconsistent with Hayashi and colleagues’ definition of food noise.

**Table 3:** Perceived purpose of TikTok videos about “Food Noise” and consistency with the theoretical definition (*n* = 99).

Variable	Category	*n*	%
Video aims to inform	No	45	45.45
	Yes	54	54.55
Video aims to entertain	No	88	88.89
	Yes	11	11.11
Video is a patient testimony	No	29	29.29
	Yes	70	70.71
Recommends or endorses a product	No	89	89.90
	Yes	10	10.10
Discloses sponsorship	No	94	94.95
	Yes	5	5.05
References scientific literature	No	94	94.95
	Yes	5	5.05
Defines food noise consistently with Hayashi et al.	No	5	5.05
	Yes	77	77.78
	No definition is provided	17	17.17
Creator’s attitude toward food noise	Positive	1	1.01
	Negative	85	85.86
	Ambivalent	1	1.01
	Neutral	12	12.12

Categories were coded based on perceived purpose and content analysis. Consistency with the theoretical definition of food noise by Hayashi et al. of “Heightened and/or persistent manifestations of food cue reactivity, often leading to food-related intrusive thoughts and maladaptive eating behaviors.” Percentages may not sum to 100% due to rounding.

The use of dances, music, and reenactments was uncommon in the videos included in the analysis, and most videos relied on text on screen (*n* = 84) and original audio from the content creator (*n* = 80) as means to communicate with viewers (Table 4).

**Table 4:** Communication strategies observed in TikTok videos about “Food Noise” (*n* = 99).

Strategy	Category	*n*	%
Text appears on screen	No	15	15.15
	Yes	84	84.85
Video shows creator	No	2	2.02
	Yes	97	97.98
Contains acting or reenactment	No	79	79.80
	Yes	20	20.20
Contains dancing	No	98	98.99
	Yes	1	1.01
Contains music or singing	No	90	90.91
	Yes	9	9.09
Audio is original	No	19	19.19
	Yes	80	80.81
Video is a stitch	No	89	89.90
	Yes	10	10.10

Categories refer to observed features within the videos. “Stitch” refers to TikTok’s feature allowing creators to respond to or incorporate clips from other videos.

Among the topics mentioned in the videos, medications and food were mentioned in 49 and 42 of the videos, respectively (Table 5). Candies and desserts were mentioned in 24 videos, and fast foods in 19. Coffee was mentioned seven times. “Fruit”, “bread and pasta”, and “meat and eggs” were mentioned six times each. Vegetables and industrialized savory snacks were mentioned five times each. Eight other food and beverage categories were mentioned two or fewer times each (*n* = 2: soda, alcohol, dairy, protein supplements, *n* = 1: sushi, peanut butter, ranch dressing, sports drinks). The frequency at which medications were mentioned in the videos was: non-specified GLP-1RAs (*n* = 17); brand-name GLP-1RA-based medications: Mounjaro (*n* = 10), Ozempic (*n* = 7), Zepbound (*n* = 4), Wegovy (*n* = 1); generic GLP-1RA molecules: tirzepatide (*n* = 7), semaglutide (*n* = 7), liraglutide (*n* = 1). Each of the following medications was mentioned once across videos: phentermine, the combination of naltrexone-bupropion, Vyvanse (brand name for lisdexamfetamine), Wellbutrin (brand name for bupropion), and naltrexone.

**Table 5:** Topics mentioned in TikTok videos about “Food Noise” (*n* = 99).

Theme mentioned in the video	*n*	%
Food	42	42.42
Medication	49	49.49
Compound medication	3	3.03
Nutritional or herbal supplement	4	4.04
Other product, service, or treatment	7	7.07
Behavioral strategy to manage food noise	16	16.16
Diet or dietary pattern	17	17.17
Body image concerns	2	2.02

Variables reflect any mention within the video. A single video might have mentioned multiple topics.

Patient testimonies on the use of medications for managing food noise (Table 6) were present in 33 videos, and testimonies on behavioral strategies were present in 12. Table 7 shows the proportion of videos that include explicit advice and recommendations to manage food noise, including behavioral strategies (*n* = 12), other products, services, or treatments (*n* = 6), food (*n* = 5), dietary patterns (*n* = 5), and medication (*n* = 4). The displayed attitude towards the use of medications to manage food noise was positive in 75.51% of videos that mentioned medications, neutral in 14.29%, ambivalent in 6.12%, and negative in 4.08% (Table 8). Table 9 contains quotes that exemplify ways in which content creators discussed the use of medications and other strategies to manage food noise.

**Table 6:** Presence of testimonies in TikTok videos about “Food Noise” (*n* = 99).

Testimony topic	*n*	%
Medication	33	33.33
Compound medication	1	1.01
Nutritional or herbal supplement	3	3.03
Other product, service, or treatment	3	3.03
Behavioral strategy to manage food noise	12	12.12
Diet or dietary pattern	10	10.10
No testimonies present	19	19.19

“Testimony” includes direct personal experiences or anecdotal narratives shared by the content creator, either verbally or through video captions. A single video might have included multiple testimonies.

**Table 7:** Advice provided by TikTok content creators about “Food Noise” (*n* = 99).

Advice topic	*n*	%
Food	5	5.05
Medication	4	4.04
Compound medication	0	0
Nutritional or herbal supplement	0	0
Other product, service, or treatment	6	6.06
Behavioral strategy to manage food noise	12	12.12
Diet or dietary pattern	5	5.05
No advice provided	63	63.64

Advice was coded as instances where creators explicitly recommended a specific strategy, treatment, or product for managing food noise. A single video might have advice on multiple topics.

**Table 8:** Creator attitudes toward treatment strategies mentioned in TikTok videos about “Food Noise”.

Treatment strategy	Positive	Negative	Ambivalent	Neutral	Total videos
Medication	37 (75.51%)	2 (4.08%)	3 (6.12%)	7 (14.29%)	49
Compound medication	3 (100%)	0	0	0	3
Nutritional or herbal supplement	4 (100%)	0	0	0	4

Percentages are calculated within each row based on the number of videos in which the treatment strategy was mentioned. Videos were coded based on tone, language, and visual cues expressing the creator’s attitude toward the treatment.

**Table 9:** Illustrative quotes from TikTok content creators discussing “Food Noise”.

Quote	Context
“And just like that, quiet. They’ve actually found something to help the chaos, to help the obsessions and compulsions.”	A content creator’s remarks after injecting themself with a GLP-1RA medication on camera
“These medications help us quiet the food noise. For the first time in my life, I have a healthy relationship with food.”	A content creator’s testimony on the use of GLP-1RAs
“[semaglutide] didn’t cure you because the food noise will be back after you stop the medication.”	A content creator’s warning about the need to continuously take semaglutide to manage food noise
“I am scared to go back (…) I’m scared that compulsive thinking is going to come back.”	A content creator expressing fear at stopping Mounjaro after finding out that their new health insurance provider refused to cover its cost
“Stimulate dopamine: get exercise, sleep well, daily sunlight, reading, listening to music, meditation.”	A content creator’s advice on behavioral strategies to manage food noise
“Taking oneself away from the kitchen or from the food cue for 10 minutes and seeing if you still feel the same. After a dog stroll, it [food noise] faded.”	A content creator’s advice on behavioral strategies to manage food noise, through the management of food cues in the environment
“Eating when hungry, not following a diet, reducing shame, not limiting food.”	A content creator’s advice on managing food noise through food and behavioral strategies
“More protein, more fiber, avoid over restricting, never skip meals, eat enough.”	A content creator’s advice on managing food noise through changes in dietary patterns

Quotes were selected to illustrate perspectives shared by TikTok content creators on strategies to manage food noise.

**Fig. 1:**
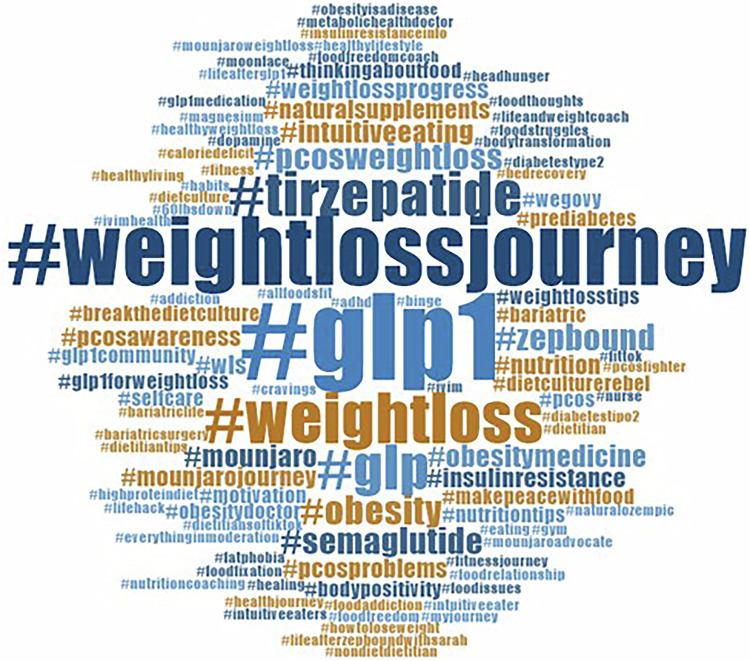
Word cloud representing indexing terms (hashtags) other than #FoodNoise included by content creators in their videos. A larger font size represents a greater frequency of a given hashtag used by content creators in addition to #FoodNoise.

A total of 143 unique hashtags were used by content creators in addition to #FoodNoise to categorize their videos within TikTok. Figure 1 visually represents their frequency. The frequencies of the top hashtags (represented in larger font) were: #GLP1 (*n* = 22), #WeightLossJourney (*n* = 17), #WeightLoss (*n* = 12), #GLP (*n* = 10), #Tirzepatide (*n* = 10), and #Obesity (*n* = 6). The Hashtags #Semaglutide #Zepbound, and #PCOSWeightLoss were used five times each. The complete list of hashtags and their frequencies is available in Appendix B.

## Discussion

In this quantitative content analysis of the top 100 videos listed by TikTok on June 24, 2024 under the hashtag #foodnoise, we examined key demographic characteristics of content creators, the way they depicted food noise, and topics associated with food noise in those videos. Most videos analyzed featured content creators who appeared to be adult females, with less than a fifth of content creators seemingly belonging to the emerging adult and none to the adolescent demographic groups. This demographic distribution is atypical for TikTok, considering that not only adolescents and emerging adults comprise most of the TikTok userbase [11, 21] and the platform’s primary target audience [22], but also make up most of TikTok’s content creators [23]. This finding is consistent with a recent analysis of 130 “MedTok” videos by Rosen et al. across 13 common health conditions in which less than half of the content creators were perceived as younger than 30 years old, and adults aged 30-49 were the largest demographic [14], suggesting that adult users are the main demographic sharing medical content on TikTok, and thus could help explain the demographic characteristics of content creators in the videos included in our analysis. Additionally, considering obesity rates in the US are higher among adults than children and adolescents, and among females than males [24], the skewed demographic characteristics of content creators in our sample of videos could be partially explained by an association between having obesity and experiencing food noise [1]. Future research, however, should examine whether food noise affects different demographic groups disproportionately, which should not be inferred based on our data, since groups might also differ in how willing they are to share their experiences on this phenomenon online. In that same paper by Rosen et al., however, 62.3% of content creators were identified as healthcare professionals [14], a much higher proportion than that observed in our sample of videos (22.2%), which was mostly comprised of patient testimonies regarding their experiences with food noise, how it affects their lives, and how they cope with it. Mentions of scientific literature to support the information provided in the videos were equally uncommon in this previous study, which is similar to the results presented here, which indicates that the practice of citing actual research is not prevalent within health-related videos shared on TikTok [14].

Foods and medications were topics mentioned in nearly half of the videos included in this analysis. Interestingly, the two categories of foods most often mentioned or depicted in conjunction with food noise were candies and desserts (which were combined into a single category), and fast foods, which are usually highly palatable, energy-dense, and highly processed. These foods are consistent with what Roe and Rolls (2021) described as “problem foods,” defined as foods that individuals find hard to resist or stop eating once they have started [25]. In their study, sweet baked items, candy, ice cream, and pizza were commonly mentioned problem foods in a sample of 186 female adult participants. This is similar to other work which used ecological momentary assessment to study food cravings and snack consumption among sixty-one female adult participants which found that participants with higher trait levels of food cravings were more prone to think about high-calorie snacks [26]. These data contribute to the notion that such foods might be commonly associated with food noise, suggesting that patients may be talking about specific categories of foods when describing this phenomenon. Additionally, energy-dense, highly palatable, and highly processed foods commonly labeled as “problem” or “forbidden” foods, such as those most often mentioned in our sample of videos, frequently evoke conflicting attitudes due to simultaneous positive (e.g., tasty, convenient, affordable, rewarding) and negative evaluations (e.g., unhealthy, ultraprocessed, source of undesired weight gain and loss of control) within the same individual [27]. The feelings of conflict that may arise from the incompatibility between health-related goals (long-term) and hedonic drives (short-term) to consume such foods lead to attitudinal ambivalence, which favors behavioral inertia–a barrier to behavior change–and could help explain the intrusive characteristics of thoughts about these foods among dieters and people trying to limit their energy intake, thus contributing to the phenomenon of food noise [27].

In the sample of videos analyzed, mentions of medications were highly prevalent, especially GLP-1RAs. This was expected, since a significant portion of anecdotal evidence available in mainstream media articles on the phenomenon of food noise is in the context of the rise in use of GLP-1RAs as anti-obesity medications supposedly capable of “silencing” food noise [1]. Content creators’ apparent attitudes towards food noise were predominantly negative, while attitudes toward using medications to manage food noise were primarily positive, as depicted in illustrative quotes transcribed from videos that highlight the relief provided by GLP-1RAs and the suffering and loss of quality of life caused by food noise. This negative view of food noise is further reinforced by the fact that over three-quarters of videos described food noise consistently with Hayashi et al.’s definition of “heightened and/or persistent manifestations of food cue reactivity, often leading to food-related intrusive thoughts and maladaptive eating behaviors,” [1] which distinguishes food noise from typical manifestations of food cue reactivity based on its heightened, persistent, and disruptive quality. Interestingly, 5.05% of videos described food noise inconsistently with the aforementioned definition. For example, one content creator described food noise as “just hunger, appetite, and craving,” challenging the theoretical assumption that food noise is an inherently negative experience. This lack of consensus among reports identified in this content analysis highlights the emerging nature of food noise and how the interpretation of its meaning might contrast across individuals. However, it is mostly consistent throughout anecdotal reports. It is important to note that, although alternative definitions for food noise have been proposed [7, 8] since Hayashi and colleagues published their narrative review exploring the construct of food noise, asserting which theoretical definition available in the scientific literature best captures definitions and examples given by content creators in their videos on TikTok was beyond the scope of this paper. Hayashi et al.’s definition was used as a reference for the present analysis because it was the only one available at the time when analyses were conducted and videos were coded. It can be argued that the criteria employed during analyses to determine consistency with Hayashi et al.’s definition of food noise focused on elements shared across all three currently available definitions (Hayashi et al.’s, Diktas et al,’s, and Dhurandhar et al.’s) [1, 7, 8], and rather than supporting any given one definition over the others, our results suggest that the majority of the videos included in our analysis seem to reflect currently hypothesized aspects of food noise. Future work is needed to fully understand how individuals utilize the term food noise in contexts beyond social media and how it can be distinguished from general reactivity to food cues that is not maladaptive.

The majority of videos analyzed did not include explicit advice or recommendations by content creators, since the majority of videos consisted of patient testimonies and personal anecdotes. However, the proportion of videos in which explicit advice was included represents about a third (36.36%) of our sample, which raises questions regarding the accuracy of the advice provided by content creators, most of which revolved around behavioral recommendations, food, and diet. Despite TikTok’s popularity in disseminating health information, numerous research studies have found conflicting data on the accuracy and credibility of the content published. For example, an analysis of the top 100 videos with the hashtag #acne found that there was a significant amount of possible shortcomings in the medical information presented [28]. Similarly, researchers assessed 225 videos about varicoceles, where they found that healthcare professionals presented medical information of “poor” quality, and non-healthcare workers presented medical information of “very poor” quality [29]. In that same study, the authors found that healthcare workers had higher viewership than non-healthcare workers. Additionally, other research examining medical information on social media has found that viewers tend to give greater credibility to content created by healthcare workers compared to non-clinician influencers [15]. Although analyzing the quality and accuracy of advice surrounding food noise provided in the videos was beyond the scope of our study, future studies exploring the quality of advice shared on social media on managing food noise would be of great value.

The recurrent mention of GLP1-RAs such as semaglutide and tirzepatide as a means of managing food noise in the videos included in this analysis warrants special consideration of why this occurs and its potential implications. On one hand, there is wide clinical plausibility to the claim that GLP-1RAs can minimize what internet users have been describing as food noise, a negatively framed preoccupation with food. Clinical studies have consistently suggested beneficial effects of GLP1RA, such as semaglutide, liraglutide, and tirzepatide, on weight loss and appetite [30–32]. The notion that these effects occur, at least in part, by managing reactivity to food cues (thus dampening food noise) is supported by experimental evidence that GLP-1RAs cause shifts in food cognition through reduced hypothalamic responses to food cues [33]. In that sense, the high prevalence of videos mentioning the use of GLP-1RAs, especially patient testimonies highlighting how these medications helped them manage their food noise, indicates that TikTok offers these individuals a community where they can share their lived experiences and express their relief at silencing intrusive thoughts about food through the use of medications that are efficient adjuvant treatments for obesity. Another factor that makes this high prevalence of patients sharing their experiences with these medications unsurprising is the sharp increase in prescriptions of GLP-1RAs in recent years, which contributes to the novelty of the topic on social media. For instance, electronic health record data from Truveta found that between January 2018 and September 2024, approximately 1.4 million patients were prescribed GLP1RA, totaling over 6 million prescriptions. Semaglutide was the most commonly prescribed first-time medication [34]. Blue Health Intelligence also reported that GLP1RA use for weight management increased significantly in recent years. From 2014 to 2021, only liraglutide (Saxenda) was available for weight loss, and users remained at fewer than 6000 annually. When Wegovy (semaglutide) was introduced, there was a dramatic rise from 5717 new users in 2020 to 120,763 users in 2023, a more than 2000% increase [35]. Similarly, healthcare analysts at IQVIA reported nearly 700,000 GLP-1 prescriptions across both obesity and diabetes in February 2024 alone, up 181% from two years prior [36]. On the other hand, it is essential to acknowledge that TikTok is a platform where most of the viewers are children, adolescents, and emerging adults, and that food noise is a relatively recent term without a clear consensus on its definition. Younger audiences, in particular, may not clearly distinguish food noise from normal hunger and appetite cues. Care must be exercised to ensure that this kind of content doesn’t negatively affect their relationship with food. For instance, vague depictions of food noise, such as “thinking too much about food,” could contribute to the Barnum effect, a cognitive bias in which individuals perceive vague, general statements as accurate descriptions of themselves [37]. This could make younger audiences prone to believing they are experiencing intrusive thoughts about food that require medical intervention. This is particularly concerning if there are financial conflicts of interest at play, which could make content creators particularly interested in convincing viewers that their food-related cognitions are pathological and that they should seek a specific service or treatment to manage them. Our finding that only 5.05% of videos contained a disclosure of sponsorship could either mean that most of the content we analyzed is unbiased and organic or that undisclosed sponsorship is prevalent, which would mean that viewers are unknowingly being advertised to by content creators endorsing products and services to manage food noise due to financial interests, which is not uncommon in social media platforms [9, 38].

Overall, the content analysis described in this paper offers important insights into the contents shared on TikTok about food noise and how they can help understand the experiences of people who experience food noise and how they cope with it. This study, however, is not devoid of limitations. The sample of 99 top videos under the hashtag #FoodNoise may not have been sufficient to capture a wider range of videos that could offer additional insights into this construct. The somewhat homogeneous demographic of content creators whose videos were included in his analysis (mostly White females aged 30 + ) also limits this study’s ability to explore diverse perspectives and describe how they might differ across different demographic groups. Likewise, all videos retrieved and included in the analysis were in the English language, which limits the scope of our analysis to English-speaking populations, raising the question of how food noise might be described and experienced differently across cultural contexts in which English is not spoken. The non-inclusion of user comments in our analyses also represents a limitation, since video comments can shed light on how users interact with the content presented on social media platforms. Furthermore, the use of a single hashtag (#foodnoise), although adequate to broadly explore the topic without pre-defining associated terms (such as using hashtags associated with GLP-1 RAs in the search), could miss out on other potential hashtags used by users in videos about food noise that don’t explicitly use the hashtag, which could be explored in future studies. Lastly, limitations inherent to the methodological approach also apply, such as the reliance on coder perceptions of content creator demographics, without direct engagement with content creators. Contacting content creators directly is a potential area for future research. Despite these limitations, our study contributes significantly to the understanding of patient lived experiences around food noise, and future research should explore such experiences in depth by employing mixed methods approaches, such as combining semi-structured interviews and metrics of food cue reactivity in individuals who report experiencing food noise to deepen the understanding of the meaning of this construct and how it impacts eating behaviors and quality of life.

In summary, the top content available on TikTok on the topic of food noise is mainly comprised of patient testimonies by female content creators aged 30 or older, who describe their experiences around food noise as negative and distressful, and depict the use of medications, mostly GLP-1RAs, as a positive strategy to help manage food noise. The way content creators describe food noise in their videos on TikTok is mostly consistent with previous literature, which suggests that it arises from intense and persistent manifestation of food cue reactivity and manifests as food-related intrusive thoughts that can contribute to maladaptive eating behaviors. Future research is needed to further understand the experiences reported by patients around food noise and how they understand and use the term beyond social media. Additionally, by refining their understanding of food noise as a determinant of human eating behavior and patient well-being, researchers and clinicians should leverage this knowledge to develop ways to assess and address food noise to improve patient outcomes.

## Supplementary information

Appendix

## Data Availability

The datasets generated and/or analyzed during the current study are not publicly available due to ethical considerations regarding the privacy of content creators whose videos were analyzed and to protect their reputational interests, but are available from the corresponding author upon reasonable request.
