# Supplementary material for: “And just like that, quiet”: a content analysis of TikTok videos on food noise
Source: Nutr Diabetes. 2026 Apr 29;16:16. doi: 10.1038/s41387-026-00423-z (PMC13265739; doi:10.1038/s41387-026-00423-z)
Supplement: Supplementary file 1 — Appendix [file 41387_2026_423_MOESM1_ESM.docx]

# Appendix

# TikTok Video Coding Form

This document is a simplified version of the RedCap form used to code the videos in this project. It contains all 17 multiple-choice questions and 10 text-input fields.

## Demographics

- Perceived age: Adolescent (13–17), Emerging adult (18–29), adult (30+)
- Perceived sex: Female, Male
- Perceived race: White, Non-White
- Healthcare professional: Yes / No
- Claimed medical profession: __________

## Sponsorship and References

- Sponsorship statement: Yes / No
- Sponsor name: __________
- Mentions scientific literature: Yes / No

## Communication Strategies

- On-screen text
- Creator appears on-screen
- Acting/reenactment
- Dance
- Music/singing
- Original audio: Yes / No
- Stitch video: Yes / No

## Purpose of the Video

- Provide information / educate
- Entertain
- Share personal testimony
- Promote product/service/treatment
- Other

## Food Noise Definition Check

- Consistent with Hayashi et al.’s definition (heightened and/or persistent manifestations of food cue reactivity, often leading to food-related intrusive thoughts and maladaptive eating behaviors): Yes / No / Not mentioned
- Transcribe definition: ______________________

## Overall Attitude Toward Food Noise

- Positive / Negative / Ambivalent / Neutral

## Topics Mentioned

- Food
- Medication
- Compound medication
- Nutritional or herbal supplement
- Other product/service/treatment
- Behavioral or coping strategy
- Diet or dietary pattern
- Body image

## Visual Mentions of Topics

- Food, Medication, Compound medication, Supplements, etc. (check all that apply)

## Testimonies Given

- Which topics above contain personal testimony? (check all that apply)

## Explicit Advice Given

- Which topics above contain explicit advice? (check all that apply)

## Content-Specific Questions (transcribe)

- Food(s) mentioned: __________
- Medication(s) mentioned: __________
- Compound medication(s) mentioned: __________
- Supplements mentioned: __________
- Other product/service mentioned: __________
- Behavioral strategies mentioned: __________
- Dietary patterns mentioned: __________

## Attitudes Toward Each Topic

- Medication: Positive / Negative / Ambivalent / Neutral
- Compound medication: Positive / Negative / Ambivalent / Neutral
- Supplements: Positive / Negative / Ambivalent / Neutral

# Table with the list of hashtags and their frequencies

| **Hashtag** | **Count** |
| --- | --- |
| #glp1 | 22 |
| #weightlossjourney | 17 |
| #weightloss | 12 |
| #glp | 10 |
| #tirzepatide | 10 |
| #obesity | 6 |
| #pcosweightloss | 5 |
| #semaglutide | 5 |
| #zepbound | 5 |
| #intuitiveeating | 4 |
| #mounjaro | 4 |
| #obesitymedicine | 4 |
| #insulinresistance | 3 |
| #mounjarojourney | 3 |
| #naturalsupplements | 3 |
| #nutrition | 3 |
| #pcosawareness | 3 |
| #pcosproblems | 3 |
| #weightlossprogress | 3 |
| #wls | 3 |
| #bariatric | 2 |
| #bodypositivity | 2 |
| #breakthedietculture | 2 |
| #dietculturerebel | 2 |
| #glp1community | 2 |
| #glp1forweightloss | 2 |
| #makepeacewithfood | 2 |
| #motivation | 2 |
| #nutritiontips | 2 |
| #obesitydoctor | 2 |
| #pcos | 2 |
| #prediabetes | 2 |
| #selfcare | 2 |
| #thinkingaboutfood | 2 |
| #wegovy | 2 |
| #weightlosstips | 2 |
| #60lbsdown | 1 |
| #addiction | 1 |
| #adhd | 1 |
| #allfoodsfit | 1 |
| #bariatriclife | 1 |
| #bariatricsurgery | 1 |
| #bedrecovery | 1 |
| #binge | 1 |
| #bodytransformation | 1 |
| #caloriedeficit | 1 |
| #cravings | 1 |
| #diabetestipo2 | 1 |
| #diabetestype2 | 1 |
| #dietculture | 1 |
| #dietitian | 1 |
| #dietitiansoftiktok | 1 |
| #dietitiantips | 1 |
| #dopamine | 1 |
| #eating | 1 |
| #everythinginmoderation | 1 |
| #fatphobia | 1 |
| #fitness | 1 |
| #fitnessjourney | 1 |
| #fittok | 1 |
| #foodaddiction | 1 |
| #foodfixation | 1 |
| #foodfreedom | 1 |
| #foodfreedomcoach | 1 |
| #foodissues | 1 |
| #foodrelationship | 1 |
| #foodstruggles | 1 |
| #foodthoughts | 1 |
| #glp1medication | 1 |
| #gym | 1 |
| #habits | 1 |
| #headhunger | 1 |
| #healing | 1 |
| #healthjourney | 1 |
| #healthylifestyle | 1 |
| #healthyliving | 1 |
| #healthyweightloss | 1 |
| #highproteindiet | 1 |
| #howtoloseweight | 1 |
| #insulinresistanceinfo | 1 |
| #intuitiveeater | 1 |
| #intuitiveeaters | 1 |
| #ivim | 1 |
| #ivimhealth | 1 |
| #lifeafterglp1 | 1 |
| #lifeafterzepboundwithsarah | 1 |
| #lifeandweightcoach | 1 |
| #lifehack | 1 |
| #magnesium | 1 |
| #metabolichealthdoctor | 1 |
| #moonface | 1 |
| #mounjaroadvocate | 1 |
| #mounjaroweightloss | 1 |
| #myjourney | 1 |
| #naturalozempic | 1 |
| #nondietdietitian | 1 |
| #nurse | 1 |
| #nutritioncoaching | 1 |
| #obesityisadisease | 1 |
| #pcosfighter | 1 |
| #pcossupport | 1 |
| #peacewithfood | 1 |
| #proffee | 1 |
| #recipe | 1 |
| #regenics | 1 |
| #regenicsjourney | 1 |
| #semaglutidecommunity | 1 |
| #semaglutidecompoundweightloss | 1 |
| #semaglutideforweightloss | 1 |
| #semaglutideinjections | 1 |
| #semaglutidejourney | 1 |
| #semaglutidetribe | 1 |
| #skincareroutine | 1 |
| #supplementsforwomen | 1 |
| #tiktokdoc | 1 |
| #tips | 1 |
| #tirzepatidecompound | 1 |
| #tirzepatidejourney | 1 |
| #type2diabetes | 1 |
| #type2diabetesreversal | 1 |
| #virtualdietitian | 1 |
| #weightlosscheck | 1 |
| #weightlosscoach | 1 |
| #weightlosscoaching | 1 |
| #weightlossjourneys | 1 |
| #weightlossmotivation | 1 |
| #weightlossstruggle | 1 |
| #weightlosstalk | 1 |
| #weightwatchers | 1 |
| #weightwatcherspartner | 1 |
| #wellness | 1 |
| #wellnessculture | 1 |
| #wellnessgoals | 1 |
| #whatimeating | 1 |
| #wieiad | 1 |
| #wlslifestyle | 1 |
| #wlssupport | 1 |
| #womenshealth | 1 |
| #worththeweight | 1 |
| #wwclinic | 1 |
| #zepboundforweightmanagement | 1 |
| #zepboundjourney | 1 |
| #zepboundwithsarah | 1 |
